# Difficult Labor; Monster; Craneotomy without Instruments

**Published:** 1858-08

**Authors:** N. L. North

**Affiliations:** Brooklyn, N. Y., Consulting Physician to the Williamsburgh Dispensary, &c.


ART. V.—*Difficult Labor; Monster; Craneötomy without Instruments.* By N. L. North, M. D., Brooklyn, N. Y., Consulting Physician to the Williamsburgh Dispensary, &c.

Mary McVay taken in labor May 13th. Had a midwife.

I was called about 5 o'clock, P. M.; found patient in second stage of labor—probably been so all day; presentation natural; parietal bones far separated, and the fontaneles large and running together.

Careless examination gave the impression that the membranes were not yet ruptured: more care, however, revealed the true condition, as the bones of the head were easily felt far separated, thin and very movable.

Labor pains had been very hard, and expulsive, so that the midwife had supposed for many hours that the child would be born immediately. The mother, whom I find consumptive, having had a "bad cough" for six months, is nearly exhausted; pains decreasing and strength failing fast; is sure she shall die; is willing to have any means resorted to to save her life. Have given ergot in large quantities with no success. Have not craneotomy instruments at hand, apply the forceps and lock them without difficulty, but the head is so soft as to afford no hold to the forceps, but yields to their pressure, and upon traction they slip off; attempt the second and third time with the same result, and then feeling it unsafe to the mother to allow enough delay to obtain the craneotomy instruments, I introduce my right hand so as to grasp a portion of the head containing a parietal bone, and by closing my hand upon it succeed in crushing or breaking it; and then by further manipulation and crushing, I manage to make the spicula of bone cut through the scalp, when an enormous quantity of sanguino-serous fluid escapes with a gush, and the hydrocephalic head is collapsed and thoroughly manageable, allowing me to grasp hold of it with so much ease as to deliver without trouble; the natural uterine contractions came on in a short time, expelling the placenta, &c., &c. The mother recovered without an untoward symptom.

Tubercular difficulty somewhat relieved after recovery from the exhaustion of the labor and its consequences.

The child was something of a rare sight. My friend Dr. O. H. Smith, examined it with me. It had a double hare lip. With the exception of two slight prominences just above the angles of the month, the upper lip, the whole middle portion of the upper jaw to the malar bones each way, and the whole of the roof of the mouth and the septum nasi were wanting; the nose itself looked like the upper lip, except a slight cartilaginous protuberance in the centre seeming to be an attempt at the formation of the septum nasi. The size of the head, as near as we could calculate from measurement after birth (it being emptied of its contents) was as follows: Occipito mental (10) ten inches; occipito frontal (8½) eight and a-half inches; bi parietal (6½) six and a-half inches; cervico-bregmatic (5) inches.

